# Socio-demographic disparities in the utilisation of general practice services for Australian children - Results from a nationally representative longitudinal study

**DOI:** 10.1371/journal.pone.0176563

**Published:** 2017-04-27

**Authors:** Lixin Ou, Jack Chen, Ken Hillman

**Affiliations:** 1 Simpson Centre for Health Services Research, South Western Sydney Clinical School, University of New South Wales, Sydney, New South Wales, Australia; 2 Ingham Institute for Applied Medical Research, New South Wales, Australia; TNO, NETHERLANDS

## Abstract

**Objective:**

This study aimed to investigate the pattern of general practice services utilization for Australian children and to examine socio-demographic disparities in general practitioner (GP) visits.

**Methods:**

We used the linked data from the nationally representative Longitudinal Study of Australian Children (LSAC) and the Medicare Australia claims data record. We used survey negative binomial and logistic regression to examine the socio-demographic factors associated with the utilisation of general practice services.

**Results:**

The average number of annual GP visits gradually declined from 7.0 at 0–1 year old to 2.4 at 5–8 years (p< .001 for trend) in the infant cohort and from 3.5 at 2–4 years to 2.0 at 9–12 years (p < .001 for trend) in the child cohort. Girls were more likely to visit GPs than boys at 0–1 year old in the infant cohort (RR = 1.06, 95%CI: 1.02–1.11) and at 2–4 years in the child cohort (RR = 1.09, 95%CI: 1.04–1.14), but there were no differences at 2–4 to 5–8 year age periods in the infant cohort and at 5–8 to 9–12 year age period in the child cohort. Children from non-English speaking background were more likely to have a greater number of GP visits compared with their counterparts from English-speaking and Indigenous background up to eight year old in both cohorts (all p < .001). Children from families with the higher socio-economic position, children without private health insurance and children living in non-metropolitan were less likely to have GP consultations in both cohorts. Fair or poor parent-rated health status was associated with greater number of GP visits.

**Conclusion:**

Socio-demographic disparities existed in the utilisation of general practice services and varied at different age periods. Family socio-economic position, private health insurance coverage and region of residence strongly associates with the utilisation disparities over all age period. Further policy interventions are called to minimise the disparities in GP utilisation for children in Australian context.

## Introduction

General practice provides person-centred, continuing, comprehensive, and co-ordinated health care for all individuals, families and communities in Australia. It also plays a vital role in prevention and health management [1]. General practitioners (GPs) are meant to be the initial port of call within the health care system. In Australia, most general practice services are paid through the Medicare scheme, a universal national health insurance funded by the federal government [2]. Approximately 80% of general practice were provided with “bulk billing”, meaning those GPs accept the Medicare benefit as full payment for a service through the Medicare scheme. In these cases, Australian residents can obtain general practice services for free.

In Australia, about 85% of Australian people visited a GP at least once per year [3]. The average number of GP visits was around 5.2 times per person per year [4]. In comparison with adults, the general practice for Australian children seemed weaker given the proportion of GP visits for children falling by 0.8% per year in contrast with the proportion of children in the total population declining by 0.3% per year [4, 5]. The Bettering the Evaluation and Care of Health (BEACH) study found that GP consultations for children aged 0–14 years represented 15.8% of all GP consultations in 1998–99 and 12.5% of all GP consultations in 2008–09 [5], in contrast, 0–14 year old children represented 20.7% of all Australian population in 1998–99 [6] and 19.1 of all Australian population in 2008–99 [7]. Furthermore, it has been reported that the annual average number of GP consultations in Australia has decreased from the highest of 5.5 per head of population in 1998–99 to a low of 4.9 per head of population in 2003–04 and the reduced attendance rate arguably raised concern regarding the equity of access to primary care [4].

Numerous studies investigated ethnic and socio-economic influences on health services utilization [8–12]. For example, a study in England found that children from a lower social class accessed GPs more compared to the higher social class [12]. Conversely, using British general household survey data, Cooper and colleagues reported no evidence showing socio-economic disparities in health care use [8]. Most of these studies used survey data that may introduce recall biases. To date, there has been lack of research investigating the socio-demographic disparities in accessing to general practice for Australian children based on objective data of health services access.

Health services utilisation is a complex process closely related to contextual and individual characteristics, as well as health attitude, health care needs, and health behaviours. Understanding health services utilisation and factors that influence the utilisation can facilitate identifying reasons for disparities in health services utilisation, consumer outcomes and for formulating policies [13]. According to the Andersen conceptual theory of health behaviour [14] which is one of the most widely used models in studying health services utilisation, inequitable access can occur when social structures (i.e., ethnicity and socio-economic status), health beliefs, and enabling resources (e.g., income or health insurance) determine the use of health services, particularly for maternal and child health. There are three components: predisposing components, enabling components, and needs components, described by Andersen’s model. The predisposing component comprises family composition, social structure and health beliefs; the enabling component includes family resources and community resources; needs component—the most immediate cause of health services use—related to the perception of illness (perceived needs for health services) and response (reported family reaction to illness or potential illness) [15].

Based on the Andersen health behaviour conceptual framework, the present study aimed to investigate (1) the average number of GP visits and the rate of having a GP visit free year (having non-attendance of GPs throughout an entire year) for Australian children following their growing periods from 0–1 to 12 year old; and (2) the association between socio-demographic background and GP visits during the same study period.

## Methods

### Study design and data source

We undertook this study using linked data between two datasets: the Medicare Australia database under the Medicare Benefit Schedule (MBS), and the nationally representative the Longitudinal Study of Australian Children (LSAC), an ongoing program initiated and funded by the Australian Government Department of Families, Housing, Community Services and Indigenous Affairs (FaHCSIA) [16]. The linkage rate from the LSAC to Medicare was 93% as only children whose parents completed a consent form for the Medicare data linkage were linked [17]. Those children whose parents did not sign the consent forms were excluded.

We drew baseline demographic data from the first wave infant cohort (3–18 months old, born in 2003–2004) and child cohort (4–5 years, born in 1999–2000) of the LSAC, respectively. The details of the LSAC design and measurement instruments have been described elsewhere [18]. Briefly, the first wave interviews of the LSAC were conducted between March and November 2004 with a two-stage stratified, clustered design. The sample frame of the LSAC was selected from the Health Insurance Commission (HIC) Medicare database that includes approximately 98% of all Australian infants and children. The sample elements were firstly stratified by state or territory and then by urban or rural status. Within each stratum, approximately one of the ten Australian postcodes was randomly included in the study as the primary sampling units to ensure proportional geographic representation. Only one child per family was recruited to the LSAC. A total of 5,107 infants and 4,983 children were recruited to the first wave of the LSAC. The response rates were 64.2% and 59.4% for the infant and child cohorts, respectively. For each participating child, written consent was obtained from parents or guardians on behalf of the children. Study ethical approval was obtained through the Australian Institute of Family Studies Ethics Committee.

### Data collection

A two-and-half-hour face-to-face interview was undertaken in the home by trained professional interviewers with the primary care-giving parent, mostly the biological mother (99.7%), but at times the biological father, step parent, adoptive parent, guardian, or someone who had a parental relationship to the child. The parents also completed a written questionnaire which was later returned. A brochure in nine languages which included information about this study was used. Apart from this brochure, an interpreter was used when required. Overall there were fifty languages involved.

The data for GP visits was extracted from the MBS record. Each record represents a benefit claim classified by the Medicare item numbers. GP consultations were identified by the item numbers 1–51, 597, 599, 601, 602, 715, 5000–5067 in the MBS records. The changes to the item numbers during the study period were taken into account [2, 19, 20]. The health assessment items introduced in 2010, e.g. 701, 703, 705, and 707 were not applicable for the study children in our analysis because these items were applied to children aged four year old only whereas all of the study children were over seven years old in 2010. The GP consultation data were then linked to the demographic data derived from the LSAC. Because the MBS records we obtained covers the time between 2002 and 2012 in this study, the linked data enable us to follow the infant cohort children from birth to 8 years old and the child cohort children from 2 to 12 years old.

### Socio-demographic variables

We followed the Andersen health behaviour model [14] to construct a theory-driven predictive model of GP utilization using key socio-demographic variables from the baseline data. The predisposing variables included the sex of study children, language/ethnicity, mother’s country/region of birth, and socio-economic position. The enabling variables consisted of private health insurance coverage and region of residence. Need variable included parent-rated overall health status of the children (very good or excellent, good, fair or poor).

Language/ethnicity of children was recorded by the interviewers using defined criteria [21]. Children from English-speaking background (ESB) were defined as those whose mothers only speak English at home. Children from non-English speaking background (NESB) were defined as those whose mothers speak a language other than English at home (excluded non-English speaking Indigenous mothers). Indigenous infants were recorded as those whose biological mother or biological father identified their infants as being of Aboriginal or Torres Strait Islander origin including Indigenous children whose mothers spoke a language other than English at home [21].

The socio-economic position was presented as a z-score calculated using a combined information measure of parental educational attainments, their income, and occupational prestige among all families [22]. We classified the socio-economic position score with four quartiles (the 1^st^ quartile indicates the lowest socio-economic group). Regions of residence was categorised by the areas of “metropolitan” and “non-metropolitan,” Metropolitan zones included capital cities and metropolitan centres with a population of 100,000 or more, and non-metropolitan zones were rural and remote areas with a population of less than 100,000 [23].

### Study outcomes

The outcome measures were (a) the average number of GP visits per annum; (b) percentage of children having a GP visit free year. Both outcome measures were presented by different age periods in each cohort. We examined the outcomes by age period: 0–1, 2–4 and 5–8 years in the infant cohort and 2–4, 5–8, and 9–12 years in the child cohort.

### Statistical analysis

Data were analysed according to survey statistical principles taking into account the design features of the LSAC. Analyses were weighted for the multistage sampling design, allowing for the unequal probabilities of selection into the sample and for no response. Rao-Scott chi-square was used to test distributional differences between subgroups and cohorts for categorical variables. We assessed a linear trend for the number of GP visits and for having a GP visit free year by treating the variable of age periods as a continuous variable after adjusted for all socio-demographic variables. We used negative binomial regression to examine the influences of socio-demographic background on the number of GP visits because the over-dispersed observations for GP visits follow negative binomial distributions. Multivariate logistic regression analysis was used to examine the association between the socio-demographic background and being GP visit free throughout an entire year. We repeated the modelling analyses for the study children at ages 0–1, 2–4, 5–8 and 9–12 years. Incidence rate ratios (IRRs) and odds ratio (ORs) were presented as results from the negative binomial regression model and the multivariate logistic regression model. Both models were under controlling for parent-rate health status. We tested interaction effects between the year and sex for each model. As we found significant interaction action effect between year and sex for both cohorts, the final models presented were stratified by both cohorts and the age periods. Statistical significance was calculated with 95% confidence intervals (CIs). All analyses were undertaken using Stata/MP 12 (StataCorp., College Station, TX).

## Results

### Demographic distributions of the study children

A total of 9,521 study children were included in the analysis, including 4,866 (51.1%) in the infant cohort and 4,655 (48.9%) in the child cohort (Table 1). Of these, half were male children; 16–17% of children were from NESB, and 4–5% of them were Indigenous children; four in five of mothers were born in Australia or New Zealand, and approximately 44% were covered by private health insurance. In addition, 66.3% of the infant cohort children and 63.4% of the child cohort children lived in a metropolitan area; and approximately 3% of children had health status rated by parents as fair or poor.

**Table 1:** Socio-demographic characteristics at baseline, 2003–04 (n = 9,521).

Characteristics	Weighted percentage	
	Infant cohort (n = 4,866))	Child cohort (n = 4,655)
**Age—year**	0–1	4–5
**Sex**		
male	51.4%	51.5%
female	48.6%	48.5%
**Language/Ethnicity**		
ESB	79.0%	78.8%
NESB	16.1%	17.4%
Indigenous	4.9%	3.8%
**Mother's country/region of birth**		
Australia & New Zealand	80.1%	77.0%
UK, US & Canada	5.0%	6.1%
Non-English Europe	1.3%	1.8%
Middle East	1.7%	2.0%
Southeast Asia	4.0%	3.8%
China (Inc. Hong Kong)	1.3%	2.0%
India, Bangladesh, Pakistan & Sri Lanka	1.6%	1.9%
Others	5.0%	5.4%
**Socio-economic position**		
1st quartile (Lowest)	28.6%	28.7%
2nd quartile	25.3%	26.0%
3rd quartile	23.6%	23.9%
4th quartile	22.6%	21.4%
**Private Health insurance holder**		
yes	44.0%	43.6%
No	56.0%	56.4%
**Region of residence**		
metropolitan	66.3%	63.4%
non-metropolitan	33.7%	36.6%
**Parent-rated health**		
very good or excellent	86.9%	87.0%
good	9.9%	10.3%
fair or poor	3.1%	2.6%

Note: ESB = English speaking background; NESB = non-English speaking background.

In the infant cohort, fair or poor health status was more likely to be reported by those infants who were female (p = .028, S1 Table), were from Indigenous origins (p< .001), whose mothers were born in Australia and New Zealand (p = .021), whose family had the lowest socio-economic positions (p = .001), who had no private health insurance coverage (p = .001), or those who lived in non-metropolitan areas (p = .039). In the child cohort, the fair or poor ratings of health status were higher in female children (p = .001, S2 Table), in children from NESB and Indigenous identities (p< .001), in children whose mothers were born in Middle East, Southeast Asia, and China (Inc. Hong Kong) (p< .001), in children living in families with the lowest socio-economic positions (p< .001), and in children without covering of private health insurance (p = .001). There was no significant difference in parent-rated health status between children living in metropolitan and non-metropolitan (p = .920).

### The utilisation of general practice services at different age periods

**Fig 1:**
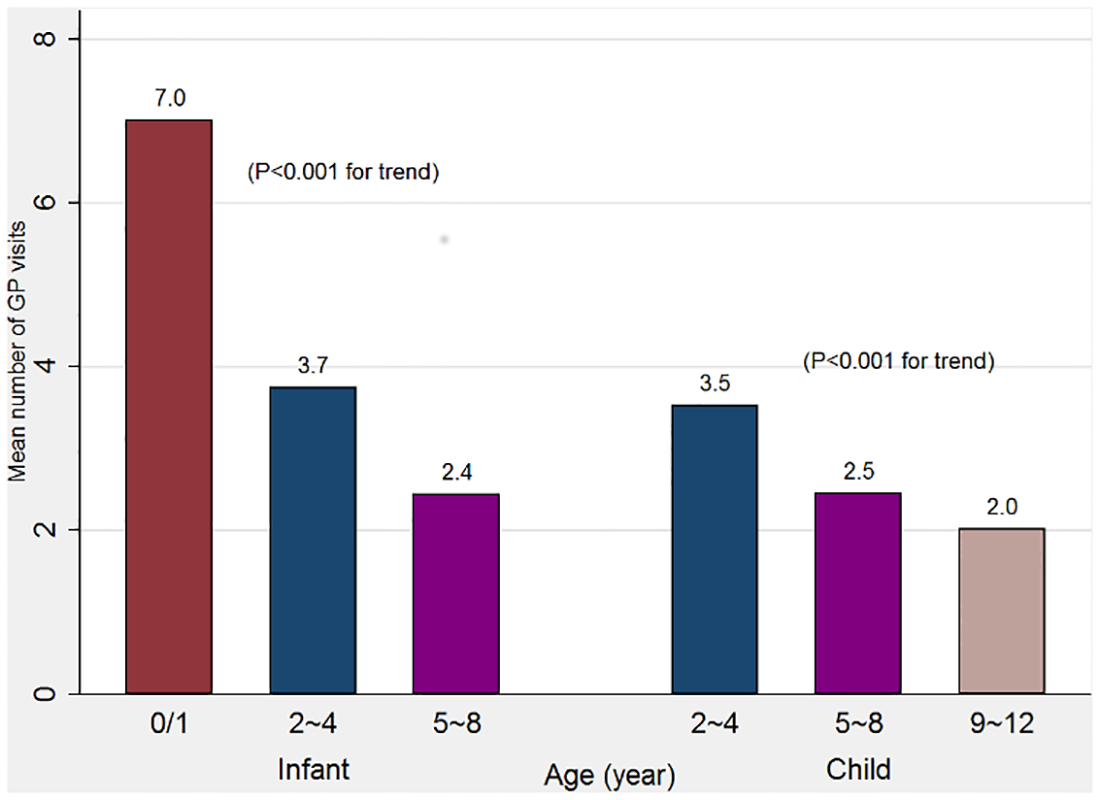
Mean number of annually GP visits for children in different age periods, by cohort.

**Fig 2:**
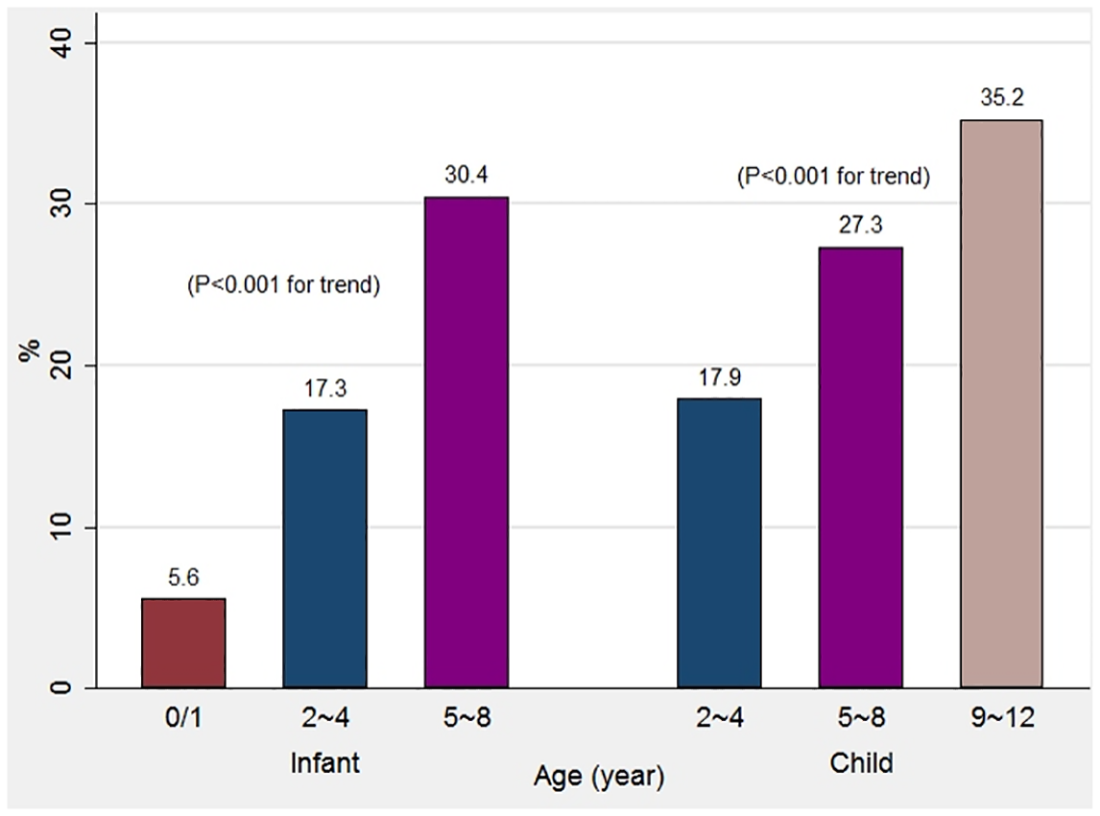
Percentage of children having a GP visit free year in different age periods, by cohort.

The mean GP visits per person per annum were 7.0, 3.7 and 2.4 for infant cohort children at ages 0–1, 2–4 and 5–8 years (p< .001 for trend) and were 3.5, 2.5 and 2.0 for child cohort children at ages 2–4, 5–8 and 9–12 years (p < .001 for trend), respectively (Fig 1). The rate of having a GP visit free year increased following children’s age periods from 5.6% at 0–1 year to 30.4% at 5–8 years in the infant cohort (p < .001 for trend) and from 17.9% at 2–4 years to 35.2% at 9–12 years in the child cohort (p < .001 for trend) (Fig 2).

### Adjusted incidence rate ratios of average number of GP visits

Among infant cohort children (0–1 year) and child cohort children (2–4 years) in 2003–2004 (Table 2), girls were more likely to visit GPs than boys (infant cohort: RR = 1.06, 95%CI: 1.02–1.1; children cohort: RR = 1.09, 95%CI: 1.04–1.14). Children from NESB were more likely to visit GP than those children from ESB and Indigenous background from birth to 8 years old (infant cohort: RR = 1.11, 95%CI: 1.03–1.21 for 0–1 year; RR = 1.22, 95%CI: 1.11–1.35 for 2–4 year; RR = 1.19, 95%CI: 1.06–1.33 for 5–8 year; child cohort: RR = 1.30, 95%CI 1.18–1.43 for 2–4 year; RR = 1.30, 95%CI: 1.16–1.46 for 5–8 year). Compared with those children whose mothers were born in Australia or New Zealand, children whose mothers were born in Middle East had more GP visits in the infant cohort, and children whose mothers were born in India, Bangladesh, Pakistan, and Sri Lanka were more likely to visit GPs in both cohorts (Infant cohort: 2–4 Yrs: RR = 1.42, 95%CI: 1.14–1.77; 5–8 Yrs: RR = 1.63, 95%CI: 1.35–1.98. Child cohort: 2–4 Yrs: RR = 1.27, 95%CI:1.09–1.49; 5–8 Yrs: RR = 1.43, 95%CI: 1.20–1.71; 9–12 Yrs: RR = 1.40, 95%CI: 1.12–1.76). Children from the families with highest socio-economic position (Infant cohort: 0–1 Yrs: RR = 0.82, 95%CI: 0.76–0.87; 2–4 Yrs: RR = 0.81, 95%CI: 0.74–0.89. Child cohort: 2–4 Yrs: RR = 0.84, 95%CI: 0.77–0.91; 5–8 Yrs: RR = 0.77, 95%CI: 0.70–0.84; 9–12 Yrs: RR = 0.86, 95%CI:: 0.78–0.94), children without private health insurance coverage (Infant cohort: 0–1 Yrs: RR = 0.94, 95%CI: 0.89–0.98; 2–4 Yrs: RR = 0.94, 95%CI: 0.88–0.99; 5–8 Yrs: RR = 0.91, 95%CI: 0.86–0.97. Child cohort: 2–4 Yrs: RR = 0.84, 95%CI: 0.79–0.89; 5–8 Yrs: RR = 0.88, 95%CI: 0.83–0.94; 9–12 Yrs: RR = 0.92, 95%CI: 0.86–0.98), and children living in non-metropolitan areas (Infant cohort: 0–1 Yrs: RR = 0.73, 95%CI: 0.69–0.77; 2–4 Yrs: RR = 0.68, 95%CI: 0.63–0.72; 5–8 Yrs: RR = 0.74, 95%CI: 0.69–0.79. Child cohort: 2–4 Yrs: RR = 0.70, 95%CI: 0.66–0.75; 5–8 Yrs: RR = 0.72, 95%CI: 0.68–0.77; 9–12 Yrs: RR = 0.80, 95%CI: 0.74–0.85) visited GPs fewer times over all age periods in both cohorts. Compared with the GP visits from children with very good or excellent parent-rated heath status, GP visits from children with fair or poor parent-rated health were 1.3–1.4 fold in the infant cohort (0–1 Yrs: RR = 1.43, 95%CI: 1.28–1.59; 2–4 Yrs: RR = 1.31, 95%CI: 1.13–1.52; 5–8 Yrs: RR = 1.36, 95%CI: 1.16–1.59), and 1.4–1.7 fold in the child cohort (2–4 Yrs: RR = 1.66, 95%CI: 1.46–1.88; 5–8 Yrs: RR = 1.67, 95%CI: 1.44–1.93; 9–12 Yrs: RR = 1.42, 95%CI: 1.20–1.67).

**Table 2:** Adjusted incidence rate ratio (95%CI) for the number of GP visits at different age periods.

Characteristics	Infant cohort			Child cohort		
	0–1 Yr	2–4 Yrs	5–8 Yrs	2–4 Yrs	5–8 Yrs	9–12 Yrs
**Sex**						
male	1.00	1.00	1.00	1.00	1.00	1.00
female	1.06** (1.02–1.11)	1.04 (0.99–1.09)	0.99 (0.94–1.05)	1.09** (1.04–1.14)	1.04 (0.99–1.09)	1.00 (0.94–1.05)
**Language/Ethnicity**						
ESB						
NESB	1.11* (1.03–1.21)	1.22** (1.11–1.35)	1.19** (1.06–1.33)	1.30** (1.18–1.43)	1.30** (1.16–1.46)	1.04 (0.91–1.18)
Indigenous	0.93 (0.84–1.03)	0.94 (0.82–1.07)	0.92 (0.79–1.07)	1.11 (0.96–1.29)	1.06 (0.91–1.23)	1.01 (0.84–1.21)
**Mother’s country/region of birth**						
Australia and New Zealand	1.00	1.00	1.00	1.00	1.00	1.00
UK, US & Canada	0.95 (0.88–1.03)	0.94 (0.84–1.04)	0.93 (0.82–1.06)	0.91 (0.82–1.01)	0.89* (0.80–0.99)	0.86** (0.77–0.96)
Non-English Europe	1.09 (0.91–1.30)	1.16 (0.90–1.50)	0.99 (0.79–1.24)	1.10 (0.93–1.30)	1.04 (0.89–1.22)	1.02 (0.82–1.25)
Middle East	1.32* (1.07–1.64)	1.32** (1.07–1.64)	1.30* (1.02–1.66)	1.06 (0.83–1.34)	1.20 (0.95–1.51)	1.47** (1.13–1.91)
Southeast Asia	1.01 (0.90–1.14)	0.95 (0.83–1.09)	0.99 (0.83–1.17)	0.97 (0.84–1.12)	1.00 (0.84–1.19)	1.02 (0.83–1.25)
China (Inc. Hong Kong)	0.82 (0.67–1.00)	1.03 (0.76–1.38)	0.91 (0.64–1.30)	1.27* (1.06–1.53)	1.14 (0.96–1.36)	1.28* (1.04–1.58)
India, Bangladesh, Pakistan & Sri Lanka	1.09 (0.95–1.25)	1.42** (1.14–1.77)	1.63** (1.35–1.98)	1.27** (1.09–1.49)	1.43** (1.20–1.71)	1.40** (1.12–1.76)
Others	1.10 (0.97–1.25)	1.07 (0.93–1.23)	1.04 (0.89–1.20)	1.00 (0.90–1.11)	1.03 (0.91–1.17)	1.06 (0.90–1.25)
**Socio-economic position**						
1^st^ quartile (lowest)	1.00	1.00	1.00	1.00	1.00	1.00
2^nd^ quartile	0.97 (0.91–1.03)	1.00 (0.92–1.08)	0.98 (0.90–1.06)	0.98 (0.91–1.05)	0.95 (0.87–1.02)	0.98 (0.91–1.07)
3^rd^ quartile	0.91** (0.86–0.97)	0.93 (0.85–1.01)	0.93 (0.84–1.02)	0.92* (0.85–1.00)	0.89** (0.82–0.97)	0.94 (0.85–1.04)
4^th^ quartile (highest)	0.82** (0.76–0.87)	0.81** (0.74–0.89)	0.81** (0.74–0.89)	0.84** (0.77–0.91)	0.77** (0.70–0.84)	0.86** (0.78–0.94)
**Private insurance coverage**						
yes	1.00	1.00	1.00	1.00	1.00	1.00
no	0.94** (0.89–0.98)	0.94* (0.88–0.99)	0.91** (0.86–0.97)	0.84** (0.79–0.89)	0.88** (0.83–0.94)	0.92* (0.86–0.98)
**Region of residence**						
metropolitan	1.00	1.00	1.00	1.00	1.00	1.00
non-metropolitan	0.73** (0.69–0.77)	0.68** (0.63–0.72)	0.74** (0.69–0.79)	0.70** (0.66–0.75)	0.72** (0.68–0.77)	0.80** (0.74–0.85)
**Parent-rated health status**						
very good or excellent	1.00	1.00	1.00	1.00	1.00	1.00
good	1.26** (1.18–1.34)	1.12* (1.03–1.22)	1.07 (0.98–1.17)	1.26** (1.17–1.37)	1.19** (1.09–1.29)	1.11* (1.01–1.23)
fair or poor	1.43** (1.28–1.59)	1.31** (1.13–1.52)	1.36** (1.16–1.59)	1.66** (1.46–1.88)	1.67** (1.44–1.93)	1.42** (1.20–1.67)

Note: ESB = English speaking background; NESB = non-English speaking background.

* *p* < .05;

** *p* < .01

### Adjusted odds ratios for having a GP visit free year

The likelihood of having a GP visit free year was lower in child cohort for 2–4 year girls (OR = 0.89, 95%CI: 0.80–0.98) and for 5–8 year children from NESB (compared to children from ESB: OR = 0.70, 95%CI: 0.57–0.85) (Table 3). However, Indigenous children who were 9–12 years were more likely to have a GP visit free year compared to their counterparts from ESB (RR = 1.26, 95%CI: 1.01–1.57). In comparison to the children whose mothers were born in Australia or New Zealand, children whose mothers were born in the UK, US and Canada were more likely to have a GP visit free year at ages 5–8 (OR = 1.20, 95% CI: 1.01–1.44) and 9–12 years (OR = 1.21, 95%CI: 1.01–1.45) in the child cohort. Those children whose mothers were born in China (including Hong Kong) were more likely to have a GP visit free year at ages 0-1(OR = 5.10, 95% CI: 2.30–11.3) and 2–4 years (OR = 2.24, 95%CI: 1.35–3.70) in the infant cohort, but less likely to have a GP visit free year at ages 9–12 years in the child cohort (OR = 0.65; 95%CI: 0.43–0.97). Conversely, infant cohort children whose mothers were born in India, Bangladesh, Pakistan, and Sri Lanka were less likely to have a GP visit free year at ages 5–8 years in the infant cohort (OR = 0.63, 95% CI: 0.42–0.94). The highest socio-economic position was associated with higher probability of a GP visit free year among infant cohort children at ages 0–1 (RR = 1.52, 95%CI: 1.00–2.31) and 2–4 years (RR = 1.23, 95%CI: 1.01–1.51), and child cohort children at ages 2–4 (RR = 1.30, 95%CI: 1.08–1.56) and 5–8 years (RR = 1.29, 95%CI: 1.12–1.50). Children without private health insurance coverage were more likely to have a GP visit free year at ages 2–4 (RR = 1.16, 95%CI: 1.01–1.33) and 5–8 years (RR = 1.23, 95%CI: 1.11–1.38) in the infant cohort, and at ages 2–4 (RR = 1.26, 95%CI: 1.12–1.43) and 9–12 years (RR = 1.16, 95%CI: 1.05–1.28) in the child cohort. Non-metropolitan residence was associated with higher rates of a GP visit free year among children at all age periods in both cohorts (Infant cohort: 0–1 Yrs: RR = 1.46, 95%CI: 1.10–1.92; 2–4 Yrs: RR = 1.54, 95%CI: 1.34–1.78; 5–8 Yrs: RR = 1.34, 95%CI: 1.20–1.50. Child cohort: 2–4 Yrs: RR = 1.63, 95%CI: 1.42–1.86; 5–8 Yrs: RR = 1.58, 95%CI: 1.41–1.77; 9–12 Yrs: RR = 1.28, 95%CI: 1.15–1.41). Fair or poor health rating was linked to the lower rates of a GP visit free year among infant cohort children at ages 5–8 years (OR = 0.69, 95%CI: 0.51–0.94) and among child cohort children at ages 2–4 (OR = 0.39, 95%CI: 0.25–0.61) and 5–8 years (OR = 0.43, 95%CI: 0.31–0.61).

**Table 3:** Odds ratio (95%CI) for GP visit free throughout an entire year at different age periods.

Characteristics	Infant cohort			Child cohort		
	0–1 Yr	2–4 Yrs	5–8 Yrs	2–4 Yrs	5–8 Yrs	9–12 Yrs
**Sex**						
male	1.00	1.00	1.00	1.00	1.00	1.00
female	0.84 (0.67–1.05)	0.92 (0.82–1.04)	1.00 (0.91–1.10)	0.89* (0.80–0.98)	0.95 (0.86–1.04)	0.98 (0.90–1.07)
**Language/Ethnicity**						
ESB	1.00	1.00	1.00	1.00	1.00	1.00
NESB	0.75 (0.45–1.24)	0.89 (0.68–1.16)	1.02 (0.82–1.26)	0.80 (0.63–1.02)	0.70** (0.57–0.85)	1.08 (0.89–1.32)
Indigenous	1.49 (0.90–2.47)	1.18 (0.86–1.62)	1.20 (0.96–1.49)	0.86 (0.64–1.14)	0.99 (0.78–1.25)	1.26* (1.01–1.57)
**Mother’s country/region of birth**						
Australia and New Zealand	1.00	1.00	1.00	1.00	1.00	1.00
UK, US & Canada	0.76 (0.45–1.29)	1.06 (0.80–1.41)	1.12 (0.88–1.42)	1.24 (0.99–1.55)	1.20* (1.01–1.44)	1.21* (1.01–1.45)
Non-English Europe	0.28 (0.035–2.22)	0.65 (0.33–1.28)	0.86 (0.53–1.40)	0.99 (0.63–1.58)	1.00 (0.70–1.41)	1.00 (0.69–1.45)
Middle East	0.52 (0.12–2.20)	0.68 (0.31–1.54)	0.81 (0.53–1.25)	0.87 (0.47–1.60)	1.04 (0.70–1.52)	0.79 (0.52–1.19)
Southeast Asia	1.36 (0.59–3.11)	1.09 (0.70–1.69)	1.27 (0.94–1.72)	1.21 (0.83–1.75)	1.13 (0.84–1.52)	0.97 (0.75–1.26)
China (Inc. Hong Kong)	5.10** (2.30–11.3)	2.24** (1.35–3.70)	1.50 (0.91–2.48)	0.95 (0.57–1.56)	0.76 (0.49–1.19)	0.65* (0.43–0.97)
India, Bangladesh, Pakistan & Sri Lanka	0.54 (0.16–1.87)	0.61 (0.35–1.07)	0.63* (0.42–0.94)	0.65 (0.39–1.09)	0.78 (0.52–1.16)	0.69 (0.44–1.08)
Others	1.41 (0.80–2.49)	1.08 (0.79–1.49)	1.02 (0.78–1.33)	1.26 (0.93–1.71)	1.22 (0.99–1.51)	1.26 (0.99–1.60)
**Socio-economic position**						
1^st^ quartile (lowest)	1.00	1.00	1.00	1.00	1.00	1.00
2^nd^ quartile	0.96 (0.69–1.33)	0.93 (0.78–1.11)	0.90 (0.79–1.03)	1.00 (0.85–1.19)	1.12 (0.98–1.28)	0.94 (0.84–1.06)
3^rd^ quartile	1.15 (0.79–1.68)	1.06 (0.88–1.26)	0.99 (0.86–1.14)	1.03 (0.87–1.22)	1.08 (0.94–1.25)	0.96 (0.83–1.10)
4^th^ quartile (highest)	1.52* (1.00–2.31)	1.23* (1.01–1.51)	1.07 (0.92–1.25)	1.30** (1.08–1.56)	1.29** (1.12–1.50)	1.00 (0.86–1.15)
**Private insurance coverage**						
yes	1.00	1.00	1.00	1.00	1.00	1.00
No	1.09 (0.81–1.46)	1.16* (1.01–1.33)	1.23** (1.11–1.38)	1.26** (1.12–1.43)	1.11 (0.99–1.24)	1.16** (1.05–1.28)
**Region of residence**						
metropolitan	1.00	1.00	1.00	1.00	1.00	1.00
non-metropolitan	1.46** (1.10–1.92)	1.54** (1.34–1.78)	1.34** (1.20–1.50)	1.63** (1.42–1.86)	1.58** (1.41–1.77)	1.28** (1.15–1.41)
**Parent-rated health status**						
very good or excellent	1.00	1.00	1.00	1.00	1.00	1.00
good	0.56* (0.35–0.90)	0.82 (0.66–1.02)	0.85* (0.73–0.99)	0.73** (0.59–0.91)	0.88 (0.74–1.03)	0.92 (0.79–1.08)
fair or poor	0.91 (0.49–1.69)	0.89 (0.64–1.24)	0.69* (0.51–0.94)	0.39** (0.25–0.61)	0.43** (0.31–0.61)	0.77 (0.58–1.02)

Note: ESB = English speaking background; NESB = non-English speaking background.

* *p* < .05;

** *p* < .01

## Discussion

In this large nationally representative longitudinal study, we found that annual GP visits peaked at 0–1 year and then declined over following age periods. We also found that close to 95% of infants (0–1 year old) accessed GPs at least once per year. Within each cohort, NESB children visited GPs more frequently at ages 0–1, 2–4 and 8 years than their ESB counterparts, but there was no such difference between Indigenous and ESB children. Children whose mothers were born in the Asian countries such as Middle East, India, Bangladesh, Pakistan and Sri Lanka showed significant differences in GP access patterns in comparison to children whose mothers were born in Australia or New Zealand. The highest socio-economic position, private health insurance uncovered and residing in a non-metropolitan area were associated with less GP visits at all age periods, and predicted a higher chance of having a GP visit free year. Children with very good or excellent parent-rated heath had fewer GP visits compared with children with fair or poor parent-rated health at all age periods in both cohorts. Our results add further weight to the previous findings of ethnic disparities reported by the survey data [8, 10, 11] and provide further evidence on the role of socio-demographic background in the utilisation of general practice services.

A previous study had revealed that 12% of 4–6 years old children did not visit a usual medical practitioner each year in the Australia Capital Territory [24]. Our study found a higher level of non-attendance rates for children at similar ages (5–8 years, 27.3–30.4% in our study), suggesting a lower GP attendance rate for Australian children at a national level. In addition, the number of GP visits for children were lower than that for the general Australian population (infants 3.9 and children 2.6 vs general population 5.2), and the rates of having a GP visit free year for children were higher in comparison with the general population (infants 20.5% and children 27.6% vs general population 15%) [3, 4].

We especially examined GP visits during the 0/1 year as this period is important in childhood development and is expected to be a high demand for access to health services [25]. We found that the number of GP visits in early childhood (0–1 year old infants) were almost 2-fold the number at older age. There may be two possible explanations. Firstly, in 2004, the Australian government implemented the ‘Strengthening Medicare’ initiative that aimed to improve access for financially disadvantaged people and children less than 16 years of age. As part of the initiative, additional incentive MBS items for GPs were introduced to encourage bulk billing in children (MBS item 10990), in particular in regional, rural and remote areas (MBS item 10991) [2]. In our study data, more than 82% of GP consultations had used these two incentive items in conjunction with the General Medical Services Table of the MBS. We found that the short-period increase in GP visits at 0–1 year were in line with the increased use of the incentive items, reflecting a short-term improvement after the introduction of the incentive scheme. These results, to some extent, support previous findings that an increase in the bulk billing rate can increase the number of GP visits and can reduce the proportion of non-attendance of GPs [26]. Secondly, the needs for regular and frequent visits in terms of maternal and child care, immunization, growth and development monitoring, nutrition and medication during the early childhood period may have contributed to the increased number of GP visits [27].

The Andersen Behaviour Model suggests a sequence of conditions contributing to the volume of services utilisation, including three determining components: 1) factors predisposing to family use of services, 2) their ability to secure services, and 3) their need for such services [15]. Since an access action starts with health need and decision to seek help, several factors could be considered as crucial for access process: parental knowledge, beliefs, and their attitudes concerning children’s symptoms [28]. Other important potential contributors may include differential geographical distributions of ethnic and Indigenous infants and socio-economic enabling factors.

Previous researches have demonstrated that major access barriers include racial or ethnic differences, language-related barriers, cultural traditions, socio-economic disadvantages, lack of health insurance coverage, and lack of available resources [29–31]. The influence of language/ethnicity on health services utilization for children has been well documented in previous studies in ethnically diverse countries, e.g. the UK, Canada and Australia [8–11]. One unique finding from our study is that compared with the ESB children, the NESB children had significantly more GP visits from birth to 8-years old. Our study findings consisted with previous studies that children whose mothers born in some Asian countries were more likely to have GP consultations in some age periods than those mothers born in Australia and New Zealand [11, 14]. The non-significant findings regarding children from China (Inc. Hong Kong) and India, Bangladesh, Pakistan & Sri Lanka may be artifacts of the small sample sizes among these sub-populations, warranting further consideration in future studies. Such results indicate that ethnic disparities in GP visits varied at different age periods. Our current study did not support previous findings from an Australian study that reported less GP access rates by NESB [10]. Our findings revealed that children from NESB had accessed GP services much more in the later years than ESB children and that there was no significant difference between Indigenous and ESB children after adjusting health status. In the previous Australian study [10], the GP access rates were based on parental recall of visiting a GP during the previous 12 months, whereas the current study used the linked medical record over an 8 to 10-year span. Therefore, the current study findings were more likely to reflect an objective assessment of the utilization of GP services among different ethnic groups.

It is worth noting that although the current study reported non-significant differences in GP access between Indigenous and ESB children after adjusting for health status, such results may be explained by the small sample size and low power for Indigenous children. However, our previous studies using the similar data had found that Indigenous children were more likely to have poor physical health outcomes, were twice likely to have a medical condition or disability, and were more likely to have medical care needs compared to their non-Indigenous counterparts [9, 32]. The current study also demonstrated that there were higher proportions of Indigenous children whose health status were fair or poor, suggesting more needs in health care. Given that equity in health services has been defined as equal access to available care for equal need, equal utilisation for equal need, and equal quality of care for all, [33–35], further research is required to examine the adequacy of GP consultations for Indigenous children.

Children from families with lower socio-economic positions have more health needs than those within higher socio-economic positions [36]. However, the nature of the relationship between socio-economic groups and health services utilization in different settings appears less certain. In the British study [8], no relationship was found between social class and the use of GP services. Our findings were consistent with an English study finding that a lower socio-economic position was associated with an increased chance of initiating a GP visit [12]. Given that poor health status often increases health needs in children from a lower socio-economic group [37], our study results suggest that the universal health care coverage in Australia and the policy intervention in encouraging bulk billing may have provided some support for those children from a lower socio-economic group to access to GP services.

On the other hand, our study showed that children without private health insurance coverage and children living in non-metropolitan had significantly less GP visits although these sub-groups of children were more likely to have poor health status that may raise more health needs. Such results suggest that the disparities still persist among children from different socio-economic backgrounds. The fact that children with better parent-rated health status visited GP fewer times may reflect that the health status and health care needs played an important part on the access of primary care and it also highlighted the importance to take such factor into account as posited by Anderson’s model when exploring the potential socio-economic disparity of access to care.

We found that children from a non-metropolitan region had a significantly less number of GP visits and a higher rate of a GP visit free year than those residing in a metropolitan area. This might be due to the lower bulk billing rate and the increased out-of-pocket cost in the rural area [38]. Our results were consistent with previous studies that regional inequities in access to GP services remain an important issue, even in the context of bulk billing Medicare [26, 38, 39]. Given that people living within rural areas are more likely to have a poorer health status [40], our findings suggest the persistence of significant unmet needs in GP services for rural children.

The strength of the current study is that we used linked data between the LSAC and the MBS over a seven year period which provided reliably estimates of socio-demographic influences on the utilisation of general practice services for Australian children. Such an approach also has the added advantage of minimising recall bias in services utilization gathered from survey data. Furthermore, our study data makes it possible to evaluate the pattern changes in GP visits for children at different age periods. The study results are broadly representative of Australian children following their age from of 0–1 year to 9–12 years, except for those living in very remote areas [18]. Our study included children from different ethnic or language backgrounds and provided detailed information regarding socio-demographic influences in each key period of growth, which may provide some useful implications to other ethnically diverse countries.

The current study also has limitations. Firstly, we used the baseline health status, socio-economic position, and region of residence to predict the GP access in a seven year period. These variables may have changed over the time, which could lead to reduced accuracy in predicting the outcomes. Secondly, our study using parent-rated overall health status as a need variable may be limited because it did not cover all the health care needs for children. In addition, our study only provided a comparison in GP access times and rates, the magnitude of the adequacy of GP visits for each subgroup of children were not examined. Further research is needed to evaluate the demand of general practice services for children from different socio-demographic background.

## Conclusion

The average number of GP visits decreased following children’s growing up. socio-demographic disparities in the utilisation of general practice services for Australian children were substantial. Family’s socio-economic position, private health insurance coverage and region of residence strongly associated with the disparities in the utilisation of general practice services. Policy initiatives are needed to monitor the disparities of GP accesses among children in ethnic, socio-economic and regional subgroups in order to reduce the disparities.

## Supporting information

S1 Table Description of health status infant cohort. (DOCX)

S2 Table Description of health status child cohort. (DOCX)

## Abbreviations

CI: confidence interval
ESB: English speaking background
FaHCSIA: Families, Housing, Community Services and Indigenous Affairs
GP: general practitioner
IRR: Incidence rate ratios
LSAC: Longitudinal Study of Australian Children
MBS: Medicare Benefit Schedule
NESB: non-English speaking background

## References

[1] Pickard JG, Tang F. Older adults seeking mental health counseling in a NORC. Research on Aging. 2009;31(6):638–60.

[2] Australian Government Department of Health and Ageing (DoHA). Medicare Benefits Schedule Book. Canberra: DoHA, 2004.

[3] Britt H, Miller GC, Charles J, Pan Y, Valenti L, Henderson J, et al General practice activity in Australia 2005–06 General practice series no. 19. AIHW Cat. no. GEP 19. Canberra: Australian Institute of Health and Welfare, 2007.

[4] Britt H & Miller GC (Eds). General practice in Australia, health priorities and policy 1998 to 2008 General practice series no. 24. AIHW Cat. no. GEP 24. Canberra: AIHW, 2009.

[5] Britt H, Miller GC, Charles J, Henderson J, Bayram C, Valenti L, et al General practice activity in Australia 1999–00 to 2008–09: 10 year data tables General practice series no. 26. AIHW Cat. no. GEP 26. Canberra: AIHI, 2009.

[6] (ABS) ABoS. AUSTRALIAN DEMOGRAPHIC STATISTICS Canberra: ABS; 2000 [cited 2017 23nd March]. http://www.ausstats.abs.gov.au/Ausstats/subscriber.nsf/0/CCE8A24866D4585FCA2568BA00131270/$File/31010_Jun%201999.pdf.

[7] (ABS) ABoS. AUSTRALIAN DEMOGRAPHIC STATISTICS Canberra: ABS; 2010 [cited 2017 23nd March]. http://www.ausstats.abs.gov.au/Ausstats/subscriber.nsf/0/CCE8A24866D4585FCA2568BA00131270/$File/31010_Jun%201999.pdf.

[8] Cooper H, Smaje C, Arber S. Use of health services by children and young people according to ethnicity and social class: secondary analysis of a national survey. Brit Med J. 1998;317(7165):1047–51. 9774288 10.1136/bmj.317.7165.1047 PMC28687

[9] Ou L, Chen J, Garrett P, Hillman K. Ethnic and Indigenous access to early childhood healthcare services in Australia: parents’ perceived unmet needs and related barriers. Australian and New Zealand Journal of Public Health. 2011;35(1):30–7. 10.1111/j.1753-6405.2010.00633.x 21299698

[10] Ou L, Chen J, Hillman K. Health services utilisation disparities between English speaking and non-English speaking background Australian infants. BMC Public Health. 2010;10(1):182. 20374663 10.1186/1471-2458-10-182 PMC2858120

[11] Saxena S, Eliahoo J, Majeed A. Socioeconomic and ethnic group differences in self reported health status and use of health services by children and young people in England: Cross sectional study. Brit Med J. 2002;325(7363):520–3. 12217992 10.1136/bmj.325.7363.520 PMC121333

[12] Saxena S, Majeed A, Jones M. Socioeconomic differences in childhood consultation rates in general practice in England and Wales: Prospective cohort study. Brit Med J. 1999;318(7184):642–6. 10066207 10.1136/bmj.318.7184.642 PMC27771

[13] Phillips KA, Morrison KR, Andersen R, Aday LA. Understanding the context of healthcare utilization: Assessing environmental and provider-related variables in the behavioral model of utilization. Health services research. 1998;33(3 I):571–96. 9685123 PMC1070277

[14] Andersen RM. Revisiting the behavioral model and access to medical care: does it matter? Journal of Health and Social Behavior. 1995;36(1):1–10. 7738325

[15] Andersen RM. Behavioral Model of Families' Use of Health Services. Chicago, IL: Centre for Health Administration Studies, University of Chicago; 1968.

[16] Sanson A, Nicholson J, Ungerer J, Zubrick S, Wilson K, Ainleyet J, et al. Introducing the Longitudinal Study of Australian Children: LSAC discussion paper No.1 Melbourn.: Australian Institute of Family Studies.; 2002 [cited 2010 19 June]. http://www.aifs.gov.au/growingup/pubs/discussion/dp1/index.html.

[17] Australian Institute of Family Studies. Longitudinal Study of Australian Children Data User Guide—May 2008. Melbourne: AIFS, 2008.

[18] Soloff C, Lawrence D, Jognstone R. LSAC Technical Paper No.1: Sample design Melbourne: Australia Institute of Family Studies; 2005 [cited 2010 19 June]. http://www.aifs.gov.au/growingup/pubs/technical/index.html.

[19] Australian Government Department of Health and Ageing (DoHA). Medicare Benefits Schedule Book. Canberra: DoHA, 2003.

[20] Australian Government Department of Health and Ageing (DoHA). Medicare Benefits Schedule Book. Canberra: DoHA, 2010.

[21] Project Operations team. The Longitudinal Study of Australia Children:Data User Guide. Melbourne: Australia Institute of Family Studies, 2007.

[22] Blakemore T, Gibbings J, Strazdins L,. Measuring the socio-economic position of families in HILDA & LSAC. ACSPRI Social Science Methodology Conference [Internet]. 2006 16 July 2010 [cited 2010 16 July]. http://old.acspri.org.au/conference2006//proceedings/.

[23] Australian Institute of Health and Welfare (AIHW). Rural, regional and remote health—A guide to remoteness classifications AIHW Cat. No. PHE 53. Canberaa: AIHW, 2004.

[24] Kljakovic M, Ciszek K, Reynolds G, Colman S. Inequality in provider continuity for children by Australian general practitioners. BMC Family Practice. 2011;12. 10.1186/1471-2296-12-106 PMC3203042 21961728

[25] Heckman JJ. Invest in the Very Young. Chicago: Ounce of Prevention Fund and the University of Chicago Harri School of Public Policy Studies, 2000.

[26] Day SE, Alford K, Dunt D, Peacock S, Gurrin L, Voaklander D. Strengthening Medicare: Will increasing the bulk-billing rate and supply of general practitioners increase access to Medicare-funded general practitioner services and does rurality matter? Australia and New Zealand Health Policy. 2005;2(1). 10.1186/1743-8462-2-18 PMC1215471 16111496

[27] Charles J, Valenti L, Britt H. Infants Encounters and management in general practice. Australian family physician. 2012;41(5):267 22696795

[28] Anderson JG, Bartkus DE. Choice of Medical Care: A Behavioral Model of Health and Illness Behavior. Journal of Health and Social Behavior. 1973;14(4):348–62. 4773924

[29] Chung PJ, Lee TC, Morrison JL, Schuster MA. Preventive care for children in the United States: Quality and barriers. Annu Rev Public Health. 2006;27:491–515. 10.1146/annurev.publhealth.27.021405.102155 16533127

[30] Kataoka-Yahiro MR, Munet-Vilaro F. Barriers to preventive health care for young children. Journal of the American Academy of Nurse Practitioners. 2002;14(2):66–72. 11892538 10.1111/j.1745-7599.2002.tb00093.x

[31] Phillips KA, Mayer ML, Aday LA. Barriers to care among racial/ethnic groups under managed care. Health Affairs. 2000;19(4):65–75. 10916961 10.1377/hlthaff.19.4.65

[32] Ou L, Chen J, Hillman K, Eastwood J. The comparison of health status and health services utilisation between Indigenous and non-Indigenous infants in Australia. Australian and New Zealand Journal of Public Health. 2010;34(1):50–6. 10.1111/j.1753-6405.2010.00473.x 20920105

[33] Culyer AJ. Equity and equality in health and health care. Journal of Health Economics. 1993;12(4):431–57. 10131755 10.1016/0167-6296(93)90004-x

[34] Culyer AJ. Need: The idea won't do-But we still need it. Social Science and Medicine. 1995;40(6):727–30. 7747207 10.1016/0277-9536(94)00307-f

[35] Whitehead M. The concepts and principles of equity and health. International Journal of Health Services. 1992;22(3):429–45. 10.2190/986L-LHQ6-2VTE-YRRN 1644507

[36] Marmot M. Achieving health equity: from root causes to fair outcomes. Lancet. 2007;370(9593):1153–63. 10.1016/S0140-6736(07)61385-3 17905168

[37] Charles J, Valenti L, Britt H. GP visits by health care card holders. A secondary analysis of data from Bettering the Evaluation and Care of Health (BEACH), a national study of general practice activity in Australia. Australian family physician. 2003;32(1–2):85–8, 94 12647666

[38] Young AF, Dobson AJ. The decline in bulk-billing and increase in out-of-pocket costs for general practice consultations in rural areas of Australia, 1995–2001. Medical Journal of Australia. 2003;178(3):122–6. 12558483

[39] Schofield DJ, Shrestha RN, Callander EJ. Access to general practitioner services amongst underserved Australians: A microsimulation study. Human Resources for Health. 2012:1. 10.1186/1478-4491-10-1 PMC3292913 22264385

[40] Australian Institute of Health and Welfare (AIHW). Rural, regional and remote health indicators of health AIHW cat. no. PHE 59. Canberaa: AIHW, 2005.

