# Supplementary material for: Socio-demographic disparities in the utilisation of general practice services for Australian children - Results from a nationally representative longitudinal study
Source: PLoS One. 2017 Apr 27;12(4):e0176563. doi: 10.1371/journal.pone.0176563 (PMC5407608; doi:10.1371/journal.pone.0176563)
Supplement: S1 Table — (DOCX) [file pone.0176563.s001.docx]

**S1 Table Description of parent-rated health status for infant cohort - no.(weighted %)**

| **Characteristics** | **Excellent/very good** | **Good** | **Fair /poor** | **P-value** |
| --- | --- | --- | --- | --- |
| **Sex** |  |  |  |  |
| male | 2085 (87.9%) | 214 (9.6%) | 62 (2.5%) | 0.028 |
| female | 2168 (86.0%) | 246 (10.2%) | 90 (3.8%) |  |
| **Language/Ethnicity** |  |  |  |  |
| ESB | 3520 (88.1%) | 335 (8.6%) | 130 (3.3%) | <0.001 |
| NESB | 562 (84.1%) | 90 (14.4%) | 9 (1.5%) |  |
| Indigenous | 171 (77.3%) | 35 (16.4%) | 13 (6.3%) |  |
| **Mother’s country of birth** |  |  |  |  |
| Australia and New Zealand | 3467 (87.2%) | 362 (9.4%) | 134 (3.4%) | 0.021 |
| UK, US & Canada | 229 (91.2%) | 16 (6.3%) | 8 (2.6%) |  |
| Non-English Europe | 51 (85.9%) | 6 (11.4%) | 1 (2.7%) |  |
| Arab | 53 (81.0%) | 8 (16.6%) | 1 (2.4%) |  |
| Southeast Asia | 143 (80.6%) | 28 (17.6%) | 3 (1.8%) |  |
| China (Inc. Hong Kong) | 45 (87.0%) | 7 (13.0%) | 0 (0.0%) |  |
| India, Bangladesh, Pakistan & Sri Lanka | 56 (76.8%) | 15 (21.6%) | 1 (1.7%) |  |
| Others | 207 (89.4%) | 18 (9.1%) | 4 (1.5%) |  |
| **Socio-economic position** |  |  |  |  |
| 1^st^ quartile (lowest) | 1019 (83.3%) | 148 (12.7%) | 46 (4.0%) | 0.001 |
| 2^nd^ quartile | 1089 (89.8%) | 90 (7.5%) | 34 (2.7%) |  |
| 3^rd^ quartile | 1076 (88.4%) | 103 (8.8%) | 35 (2.8%) |  |
| 4^th^ quartile (highest) | 1059 (86.9%) | 117 (10.2%) | 37 (2.9%) |  |
| **Private insurance coverage** |  |  |  |  |
| yes | 2008 (89.1%) | 181 (8.2%) | 65 (2.7%) | 0.001 |
| no | 2243 (85.3%) | 278 (11.3%) | 87 (3.5%) |  |
| **Region of residence** |  |  |  |  |
| metropolitan | 2654 (87.2%) | 300 (10.3%) | 79 (2.6%) |  |
| non-metropolitan | 1599 (86.5%) | 160 (9.3%) | 73 (4.2%) | 0.039 |

Note: ESB=English speaking background; NESB=non-English speaking background.
