# Supplementary material for: Socio-demographic disparities in the utilisation of general practice services for Australian children - Results from a nationally representative longitudinal study
Source: PLoS One. 2017 Apr 27;12(4):e0176563. doi: 10.1371/journal.pone.0176563 (PMC5407608; doi:10.1371/journal.pone.0176563)
Supplement: S2 Table — (DOCX) [file pone.0176563.s002.docx]

**S2 Table Description of parent-rated health status for Child cohort - no.(weighted %)**

| **Characteristics** | **Excellent/very good** | **Good** | **Fair /poor** | **P-value** |
| --- | --- | --- | --- | --- |
| **Sex** |  |  |  |  |
| male | 2030 (89.0%) | 204 (9.1%) | 41 (1.9%) | 0.001 |
| female | 2047 (85.2%) | 261 (11.5%) | 71 (3.3%) |  |
| **Language/Ethnicity** |  |  |  |  |
| ESB | 3362 (89.0%) | 324 (8.8%) | 78 (2.2%) | <0.001 |
| NESB | 575 (79.4%) | 116 (16.3%) | 28 (4.4%) |  |
| Indigenous | 140 (81.7%) | 25 (14.4%) | 6 (3.9%) |  |
| **Mother’s country of birth** |  |  |  |  |
| Australia and New Zealand | 3196 (88.3%) | 327 (9.3%) | 83 (2.5%) | <0.001 |
| UK, US & Canada | 267 (89.3%) | 25 (8.7%) | 5 (1.9%) |  |
| Non-English Europe | 78 (93.5%) | 6 (6.5%) | 0 (0.0%) |  |
| Arab | 54 (79.1%) | 11 (16.3%) | 3 (4.6%) |  |
| Southeast Asia | 125 (76.1%) | 25 (17.4%) | 9 (6.5%) |  |
| China (Inc. Hong Kong) | 58 (75.3%) | 18 (21.2%) | 3 (3.5%) |  |
| India, Bangladesh, Pakistan & Sri Lanka | 60 (66.2%) | 25 (32.0%) | 2 (1.8%) |  |
| Others | 207 (87.0%) | 24 (10.0%) | 6 (2.9%) |  |
| **Socio-economic position** |  |  |  |  |
| 1^st^ quartile (lowest) | 957 (82.2%) | 160 (14.0%) | 44 (3.8%) | <0.001 |
| 2^nd^ quartile | 1042 (90.1%) | 98 (8.2%) | 20 (1.7%) |  |
| 3^rd^ quartile | 1033 (88.2%) | 104 (9.2%) | 24 (2.6%) |  |
| 4^th^ quartile (highest) | 1035 (88.5%) | 101 (9.2%) | 24 (2.3%) |  |
| **Private insurance coverage** |  |  |  |  |
| yes | 1943 (89.4%) | 174 (8.3%) | 44 (2.4%) | 0.001 |
| no | 2127 (85.2%) | 290 (12.0%) | 68 (2.9%) |  |
| **Region of residence** |  |  |  |  |
| metropolitan | 2517 (86.9%) | 284 (10.5%) | 68 (2.7%) |  |
| non-metropolitan | 1560 (87.3%) | 181 (10.2%) | 44 (2.6%) | 0.920 |

Note: ESB=English speaking background; NESB=non-English speaking background.
